# Distinct aspects of frontal lobe structure mediate age-related differences in fluid intelligence and multitasking

**DOI:** 10.1038/ncomms6658

**Published:** 2014-12-18

**Authors:** Rogier A. Kievit, Simon W. Davis, Daniel J. Mitchell, Jason R. Taylor, John Duncan, Cam-CAN Research team, Lorraine K. Tyler, Carol Brayne, Ed Bullmore, Andrew Calder, Rhodri Cusack, Tim Dalgleish, Fiona Matthews, William Marslen-Wilson, James Rowe, Meredith Shafto, Karen Campbell, Teresa Cheung, Linda Geerligs, Anna McCarrey, Kamen Tsvetanov, Nitin Williams, Lauren Bates, Tina Emery, Sharon Erzinçlioglu, Andrew Gadie, Sofia Gerbase, Stanimira Georgieva, Claire Hanley, Beth Parkin, David Troy, Jodie Allen, Gillian Amery, Liana Amunts, Anne Barcroft, Amanda Castle, Cheryl Dias, Jonathan Dowrick, Melissa Fair, Hayley Fisher, Anna Goulding, Adarsh Grewal, Geoff Hale, Andrew Hilton, Frances Johnson, Patricia Johnston, Thea Kavanagh-Williamson, Magdalena Kwasniewska, Alison McMinn, Kim Norman, Jessica Penrose, Fiona Roby, Diane Rowland, John Sargeant, Maggie Squire, Beth Stevens, Aldabra Stoddart, Cheryl Stone, Tracy Thompson, Ozlem Yazlik, Dan Barnes, Marie Dixon, Jaya Hillman, Joanne Mitchell, Laura Villis, Richard N.A. Henson

**Affiliations:** 1 MRC Cognition and Brain Sciences Unit, 15 Chaucer Road, Cambridge CB2 7EF, UK; 2 Department of Psychology, University of Cambridge, Downing Street, Cambridge CB2 3EB, UK; 3 School of Psychological Sciences, The University of Manchester, Brunswick Street, Manchester M13 9PL, UK; 4 Cambridge Centre for Ageing and Neuroscience (Cam-CAN), University of Cambridge and MRC Cognition and Brain Sciences Unit, Cambridge CB2 7EF, UK

## Abstract

Ageing is characterized by declines on a variety of cognitive measures. These declines are often attributed to a general, unitary underlying cause, such as a reduction in executive function owing to atrophy of the prefrontal cortex. However, age-related changes are likely multifactorial, and the relationship between neural changes and cognitive measures is not well-understood. Here we address this in a large (*N*=567), population-based sample drawn from the Cambridge Centre for Ageing and Neuroscience (Cam-CAN) data. We relate fluid intelligence and multitasking to multiple brain measures, including grey matter in various prefrontal regions and white matter integrity connecting those regions. We show that multitasking and fluid intelligence are separable cognitive abilities, with differential sensitivities to age, which are mediated by distinct neural subsystems that show different prediction in older versus younger individuals. These results suggest that prefrontal ageing is a manifold process demanding multifaceted models of neurocognitive ageing.

## Abstract (web summary)

Executive functions are affected by ageing but it is unclear how. Kievit *et al.* use brain imaging and modelling approaches to show how multifactorial explanations outperform single-factor models when relating age-related differences in grey and white matter to differences in fluid intelligence and multitasking.

## Graphical abstract

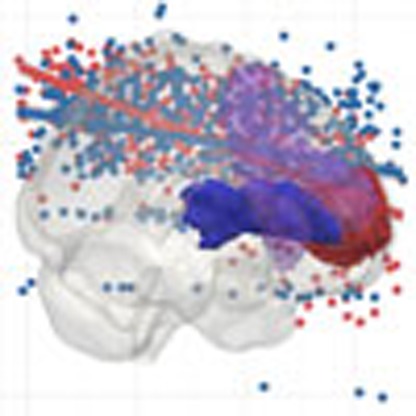

Age-related changes are widespread, but not homogeneous in terms of cognitive function or brain substrates. Executive functions and frontal brain regions, for example, show disproportionately strong age-related declines^1^,^2^ compared to other cognitive functions. These declines have far-reaching consequences for successful day-to-day functioning in old age^3^. This is concerning, given that a rapidly ageing population in western countries (a 50% increase in over 65’s expected by 2035 (ref. 4)) engenders an increasing demand for high-functioning older adults. Together, these issues present an urgent challenge for the cognitive neuroscience of ageing: how exactly do higher cognitive functions differ across the lifespan, and how are they related to concurrent neural changes?

Prominent theories of age-related cognitive decline include models that focus on a single cause or factor that is posited to influence a wide range of cognitive functions. Many one-factor models of aging suggest that declines in cognition represent a continuum, where the pathological neurocognitive extremes (for example, Alzheimer’s disease) represent the ends of that continuum^5^. This unitary view has often focused on a set of complex cognitive operations grouped under the label of executive function*s*. Although a precise definition has proved elusive, executive functions are commonly considered to include abilities such as ‘choosing and initiating goal-directed behaviours’, ‘set shifting’ and ‘cognitive flexibility’^2^, all of which show steep age-related declines relative to other cognitive functions. Concurrent decline in grey matter (GM)^1^ and white matter integrity (WMI)^6^ of the frontal lobe has been observed in longitudinal samples, leading to an influential theory of ageing known as the frontal lobe hypothesis^7^,^8^, in which declines in executive functions are attributed to age-related changes in the frontal lobe. However, the precise nature of age-related changes within the frontal lobe, such as the connection between GM and WM changes, remains unclear. This issue is key, as improved understanding of the effects of frontal atrophy on cognitive performance is necessary to develop age-related interventions that may preserve executive function into old age.

In this paper, we estimate the unique contributions of age-related differences in frontal GM and WM structures for two executive functions: fluid intelligence and multitasking. Fluid intelligence has been defined as the ability to think logically and solve problems in the absence of task-specific knowledge or experience^9^. Fluid intelligence lies at the core of psychometric analyses of intelligence^9^ and predicts real world outcomes including life expectancy, expected income and work performance^10^. Moreover, fluid intelligence correlates highly with tests that assess successful day-to-day functioning in society such as the Basic Skills Test^11^, even across intervals of several years^3^. Multitasking, on the other hand, is the general ability to perform several tasks simultaneously without adverse effects on performance. Lower multitasking ability is associated with a history of other age-related challenges such as recurrent falls^12^ and possibly underlies age-related problems on other cognitive abilities such as working memory^13^.

More generally, behavioural tests of executive functions are likely to share certain cognitive components, and are generally positively correlated^14^. They therefore represent a valuable avenue of insight into models of age-related changes in executive function: They allow for comparison of single versus multifactorial models, and an examination of distinct contributions of different neural properties of the frontal lobe. Our goal here is not to exhaustively cover the domain of executive functions, but instead to focus on two specific tests that are likely to measure dissociable executive functions (fluid intelligence and multi-tasking)—to put to test the prevalent unidimensional theories of executive function in ageing.

More specifically, our goals are to examine whether age-related differences in these executive functions are unitary or multifactorial; whether the dependence on frontal lobe differences is global or specific and to what extent neural differences explain age-related differences in these cognitive factors. In a large (*N*=567) age-heterogeneous population-based sample from the Cambridge Centre for Ageing and Neuroscience (Cam-CAN), we use (confirmatory) structural equation modelling (SEM), a powerful multivariate technique that fits observed covariances between variables^15^, and allows for the joint testing of several *a priori* predictions. Structural equation modelling has been used to model age-related differences in cognition, and is increasingly applied to capture cognitive and neural differences across lifetime^16^,^17^,^18^,^19^,^20^,^21^,^22^. Using these techniques, we show how age-related differences in fluid intelligence and multitasking are related to distinct measures of frontal lobe integrity.

The challenge in any neuroscientific study of ageing is how to best assess and interpret age-related differences^23^. As extant models of aging rely largely on age-heterogeneous cross-sectional data, it is important to keep in mind that such studies likely reflect a combination of intra-individual decline and inter-individual cohort effects (for example, year of birth). Methodological approaches have focused on the challenges for age-heterogeneous cross-sectional designs^23^,^24^,^25^. Others have defended cross-sectional studies^26^,^27^, arguing that longitudinal studies also suffer from inferential challenges, including underestimation of true ageing effects due to practice effects^28^ and selective attrition^27^,^29^.

Madden *et al.*^30^ proposed three heuristic criteria for interpreting brain–behaviour relationships in cross-sectional neurocognitive ageing studies. First, the relationship between brain and behaviour should be significant when age is taken into account, to ensure that the cross-sectional correlation is not merely due to co-occurrence of decline^31^. Second, the age-to-cognition relationship should be attenuated when age-associated neural differences are taken into account^19^. Finally, to establish if there is a difference in the brain–behaviour relationship for young and old, this difference should be formally tested, as opposed to comparing significant versus non-significant relationships in the young and old. We will show that the data reported here satisfy all three criteria.

We focus on four neural properties selected based on the current literature—(see Methods), two involving grey matter volume (GMV; Brodmann Area 10 (BA10) and the Multiple Demand (MD) System), and two involving WMI (the Forceps Minor (FM) and the Anterior Thalamic Radiations (ATR)). Fitting a series of structural equation models, we show that multitasking and fluid intelligence are distinct cognitive abilities that show diverging age-related differences, and are mediated by distinct neural subsystems and show differential brain–behaviour patterns in older versus younger individuals. These results show that the relation between age-related differences in executive function and lifetime changes in neural structure is multidimensional. Principled application of formal statistical models will further increase understanding of these processes.

## Results

### Competing factor models of brain and behavioural differences

**Figure 1:**
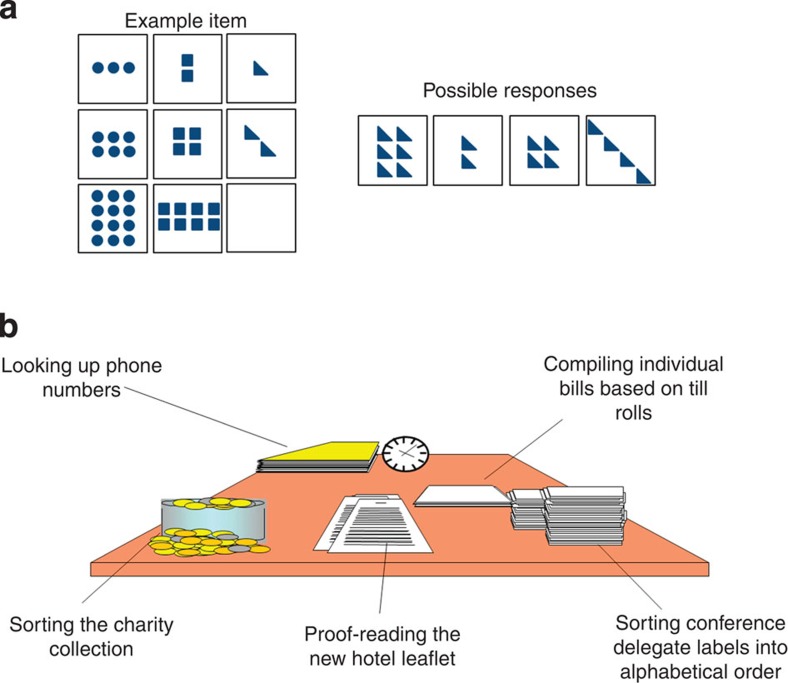
Fluid intelligence and multitasking tasks. (**a**) Shows an example of a fluid reasoning item (trial). The Cattell test yields scores on four subtests used for further modelling. (**b**) Shows the multitasking task, which is a simulation of a hotel environment, in which participants were asked to perform each of the five tasks for equal amounts of time within a 10-min period. Variables-of-interest are the number of different tasks people performed and total time misallocated.

Following best practice in SEM^32^, we first report our measurement model on the full sample of *N*=567 (for further details on the sample and model fitting, see Methods). For this model, we hypothesize that two latent variables (fluid intelligence and multitasking; tasks shown in Fig. 1, see Methods for more detail) capture the covariance between the six behavioural variables described in the Methods section, freely estimating every factor loading. This model fits the data well, *χ*^2^=15.40, degrees of freedom (df)=8, *P*=0.052, root mean square error of approximation (RMSEA)=0.04 (0.00–0.070), comparative fit index (CFI)=0.993, standardized root mean square residual (SRMR)=0.023, Satorra–Bentler scaling factor=1.009. As the two latent factors are positively correlated (standardized estimate=0.325, *Z*=6.17, *P*<0.001), we can ask whether a more parsimonious model with only a single cognitive factor (for example, ‘executive function’) shows better fit. Such a model would be compatible with a unitary perspective on the age-related decline of higher cognitive function. However, this one-factor model fits very poorly: *χ*^2^=334.149, df=9, *P*<0.00001, RMSEA=0.252 (0.231–0.275), CFI=0.685, SRMR=0.121, Satorra–Bentler scaling factor=1.109, significantly worse than the two-factor model (*χ*^2^_diff_=46.224, df_diff_=1, *P*<0.00001). One additional alternative, a hierarchical model with a third, higher-order latent variable, fits the data equivalently to the single-factor model above, but has one extra parameter, so the more parsimonious model is preferred (AIC_diff_=2).

**Figure 2:**
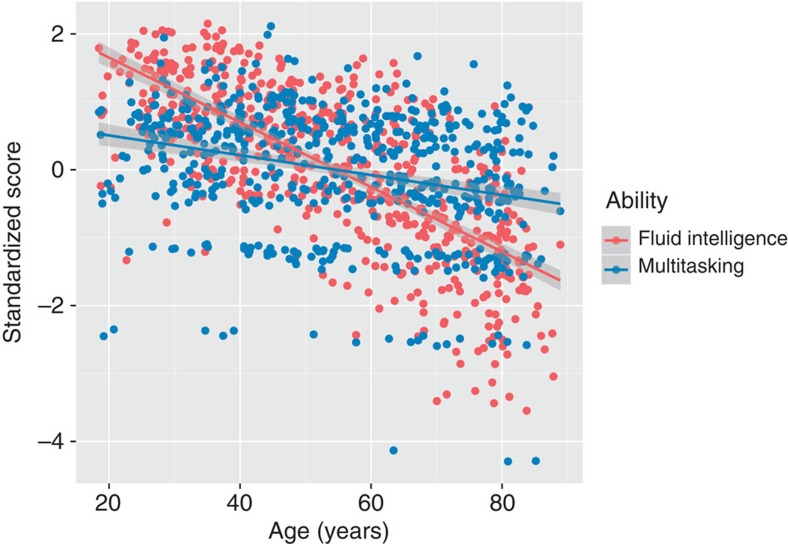
Linear fit of fluid intelligence and multitasking with age. Fluid intelligence shows a significantly stronger age-related difference (*r*=−0.67) than multitasking (*r*=−0.29) (William’s test for dependent (*r*=0.38) correlations *t*(564)=10.66, *P*<0.00001).

Together these findings suggest that multitasking and fluid intelligence are separable cognitive factors. We subsequently estimate the impact of age on both cognitive factors. Figure 2 shows that although both factors differ significantly with age, scores on the latent variable of fluid intelligence decline significantly more quickly (*r*(565)=−0.67, *P*<0.0001, 95% confidence interval (CI): (−0.71,−0.61)) than scores on multitasking (*r*(565)=−0.29, *P*<0.0001, 95% CI: (−0.35,−0.20)): William’s test for dependent correlations sharing a variable: *t*(564)=10.66, *P*<0.00001.

There is a small but significant increase in inter-individual variability with age on both latent variables (age and fluid intelligence: Breusch–Pagan test *χ*^2^=15.4876, df=1, *P*<0.0001; age and multitasking: Breusch–Pagan test *χ*^2^=17.0027, df=1, *P*<0.0001). To ensure that the significant increase in residuals does not affect our estimates of age-related decline in fluid intelligence and multitasking, we estimate both a traditional parametric regression and a heteroscedasticity-consistent robust sandwich estimator. As can be seen in Table 1, the parameter estimates and s.e. are virtually identical, suggesting no adverse effects of the heteroscedastic residuals.

**Figure 3:**
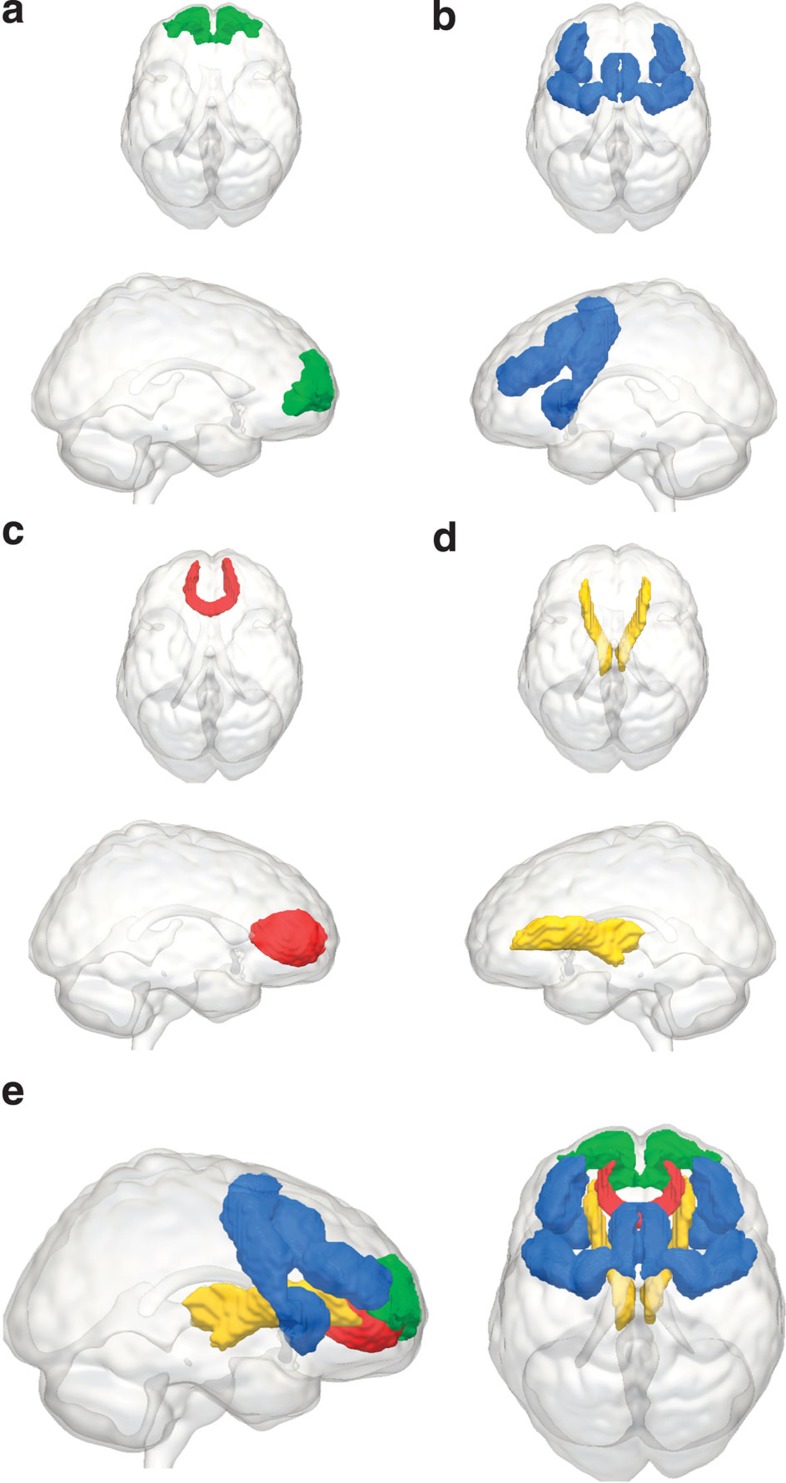
Four frontal regions-of-interest. The four neural structures-of-interest: (**a**) Brodmann Area 10 (BA10), (**b**) the Multiple Demand (MD) system, (**c**) the Forceps Minor (FM), passing through the genu of the corpus callosum, (**d**) the Anterior Thalamic Radiations (ATR) and (**e**) all four structures superimposed.

Having established the proper fit of our measurement model, we then relate these factors to the four neural variables based on the literature (see Methods for more details). These four neural variables are GMV in the frontopolar cortex (BA10), GMV in the MD system, WMI of the FM and WMI of the ATR, as shown in Fig. 3.

**Figure 4:**
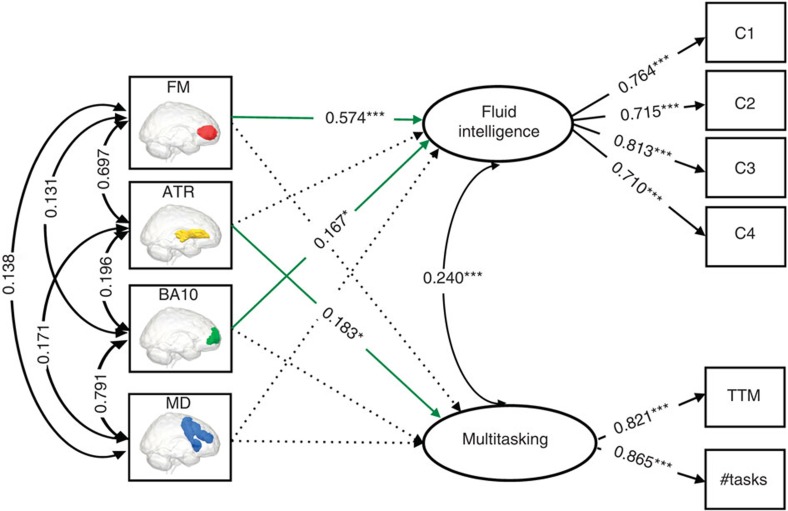
Full MIMIC model relating four frontal brain variables to fluid intelligence and multitasking. Significant brain–behaviour parameters are shown in green solid lines. All parameter estimates shown are fully standardized. This model fits the data well, *χ*^2^=52.912, *df*=24, *P*=0.001, RMSEA=0.046 (0.029–0.063), CFI=0.979, SRMR=0.029, Satorra–Bentler scaling factor=1.028. C1–C4 refer to the four sub-scores on the Cattell test of fluid intelligence; TTM (total time misallocated) and #tasks (number of tasks attempted) are two indices of performance on Hotel test of multitasking. *P<0.05, ***P<0.001. These *P*-values are based on a z-test statistic derived from a Maximum Likelihood Structural Equation Model using robust standard errors and a Satorra–Bentler scaled test statistic. See ref. 6767 for further details.

Next, we fit the full model relating behavioural variables to the brain variables (for the full covariance matrix, see Supplementary Table 1) using a type of SEM called a Multiple Indicators, Multiple Causes model (MIMIC model^33^,^34^, see Methods for further details). This model captures the hypothesis that individual differences in the two cognitive factors are causally dependent on the combination of neural properties in the prefrontal cortex (PFC). The full model, shown in Fig. 4, fits the data well: *χ*^2^=52.912, df=24, *P*=0.001, RMSEA=0.046 (0.029–0.063), CFI=0.979, SRMR=0.029, Satorra–Bentler scaling factor=1.028. The good fit of the full model allows us to further investigate the relations between the cognitive factors and the neural variables.

First and foremost, the model shows clear diverging predictions within PFC: GMV in BA10 and WMI of the FM strongly predict individual differences in fluid intelligence but not in multitasking, whereas WMI of the ATR predicts performance on multitasking, but not fluid intelligence. Contrary to our expectations, GMV in the MD system does not predict fluid intelligence above and beyond GMV of BA10. We formally test the equality of the neural parameters using a *χ*^2^-test between the fit of the full model and that of another model where the brain–behaviour parameters are constrained to be equal for fluid intelligence and multitasking. This comparison shows that the model in which the brain parameters are freely estimated fits the data significantly better than the model in which they are constrained to be equal (*χ*^2^_diff_=79.914, df=4, *P*<0.0001), suggesting that specific prefrontal brain properties have different impacts on the cognitive factors.

To further quantify these relationships, we regress the behavioural factor scores on the respective neural variables. This shows that BA10 and FM together strongly predict individual differences in fluid intelligence: adjusted *R*^2^=0.471, *F*(2, 564)=253.4, *P*<0.00001. The relationship between multitasking and ATR is less strong but also pronounced, adjusted *R*^2^=0.074, *F*(1, 546)=46.21, *P*<0.00001. To test the specificity of the four neural variables shown in the model in Fig. 4, we fit an additional model shown in Supplementary Materials (Supplementary Fig. 1). For this analysis, we include two additional control measures (GMV in the temporal pole and WMI in the forceps major) hypothesized to not predict either fluid intelligence or multitasking. In line with this hypothesis, these control measures did not have significant influences on the latent variables and, unlike the neural variables-of-interest described above, fixing their parameters to zero does not adversely affect model fit.

**Figure 5:**
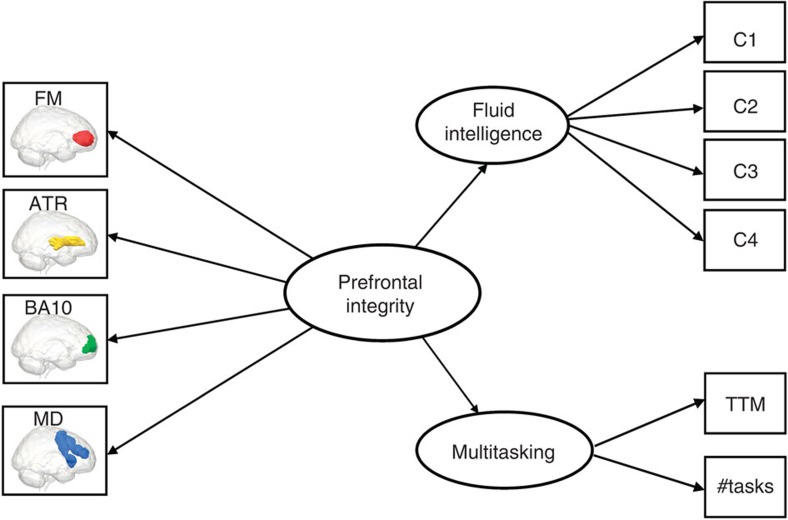
Frontal lobe model. This model represents the hypothesis that age-related individual differences in frontal lobe structure can be captured by a single factor, representing overall PFC integrity. This model fits the data poorly, *χ*^2^=670.11, df=32, *P*<0.00001, RMSEA=0.188 (0.176–0.200), CFI=0.728, SRMR=0.158, Satorra–Bentler scaling factor=1.05).

The above analyses demonstrate clear differential predictions of the GM and WM variables-of-interest. However, we can also formally test whether a simpler, one-factor model representing ‘general PFC integrity’ can capture the covariance between these frontal lobe measures. This ‘frontal lobe’ model, shown in Fig. 5, can be seen as representing the frontal lobe hypothesis, where the overall health of the PFC is a global property of a person, which determines their level of (preserved) executive function. However, this global PFC model shows poor fit, *χ*^2^=670.11, df=32, *P*<0.00001, RMSEA=0.188 (0.176–0.200), CFI=0.728, SRMR=0.158, Satorra–Bentler scaling factor=1.050, suggesting that structural brain properties of the PFC cannot be captured by a single factor.

**Figure 6:**
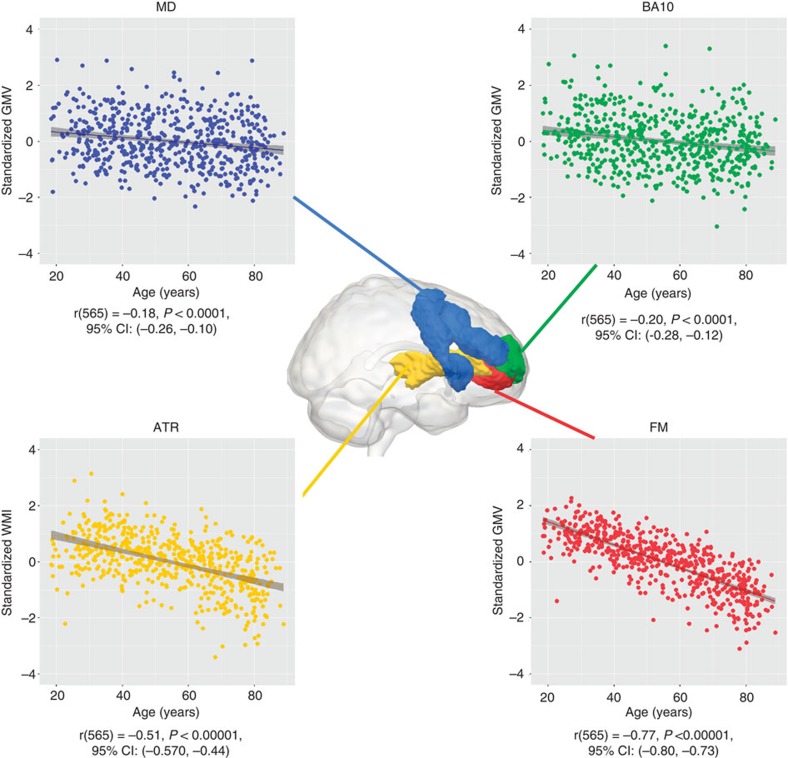
Differential prefrontal ageing. Plots show differential ageing patterns of the four PFC structures-of-interest. Forceps minor shows greatest age-related differences, followed by the anterior thalamic radiations. The two GMV regions-of-interest show equal age-related differences. This pattern further supports differentiation within the PFC.

We fit a second control model, where the two types of neural property—grey matter (GMV) and white matter (WMI)—are separate latent variables, each of which affects fluid intelligence and multitasking. This ‘neural latent variable’ model, shown in Supplementary Fig. 2, fits the data better than the frontal lobe model described above, but considerably worse than the MIMIC model shown in Fig. 4 (AIC_diff_=41.915). Taken together, these comparisons show that although global factors like GMI or WMI are known to predict important general aspects of aging^16^, they may miss more fine-grained details in the specific brain regions/tracts involved. This differentiation within the PFC is further exemplified by closer inspection of age-related differences in each of the neural variables, as can be seen in Fig. 6. The GM variables MD and BA10 show similar age-related differences (age and BA10: *r*(565)=−0.20, *P*<0.0001, 95% CI: (−0.28,−0.12), MD and age: *r*(565)=−0.18, *P*<0.0001, 95% CI: (−0.26,−0.10)), William’s test for dependent correlations sharing a variable: *t*(564)=0.7, *P*>0.4). However, WMI averaged across ATR and FM shows significantly steeper decline than GMV averaged across BA10 and MD (*t*(564)=11.84, *P*<0.00001), and WMI in FM in turn shows significantly steeper decline than WMI in ATR (*r*(565)=−0.77, *P*<0.00001, 95% CI: (−0.80,−0.73)) than WMI in ATR (*r*(565)=−0.51, *P*<0.00001, 95% CI: (−0.57,−0.44)), William’s test for dependent correlations sharing a variable: (*t*(564)=13.42, *P*<0.00001). Together, these results show that there is clear differential ageing within PFC, and that this has different consequences for specific cognitive factors.

### Mediation of age-related differences in cognition

Having shown that individual differences in fluid intelligence and multitasking can be predicted by specific brain measures, we next examine whether age-related differences in these measures might explain concurrent age-related differences in the two cognitive factors. To this end, we use mediation analysis, commonly used to study neurocognitive ageing^19^. Mediation models hypothesize three paths, a, b and c, between three variables-of-interest, and that taking into account the indirect paths (a and b) should attenuate the key relationship (c, in this case the relationship between age and cognition). We fit mediation models within a SEM framework that simultaneously models all paths, giving more powerful, accurate and robust estimation of mediation effects^35^ than more traditional tests based on sequential regressions. As noted above, we here model age-related differences in brain structure as a partial cause of the concurrent age-related differences in the two cognitive dimensions, which is not identical to observing concurrent age-related decline (rates of change). To ensure that possible mediation effects are not solely due to concurrent age-related decline of brain and behaviour (which may create artificial patterns of mediation^31^,^36^), we first examine the partial correlations between brain and behaviour after controlling for age. This shows that the correlations between fluid intelligence and BA10 (*r*(564)=0.251, *P*<0.0001), between fluid intelligence and FM (*r*(564)=0.221, *P*<0.0001) and between multitasking and ATR (*r*(564)=0.161, *P*<0.001) are all significant when controlling for age.

**Figure 7:**
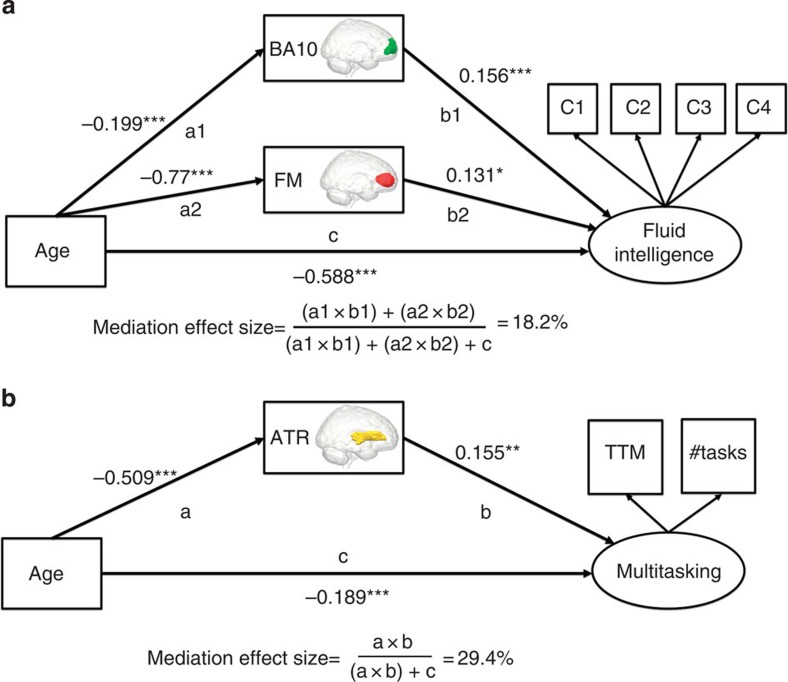
Mediation models. (**a**) Mediation model for fluid intelligence. (**b**) Mediation model for multitasking. In both models, the brain structures-of-interest significantly mediate the age-related differences in cognitive abilities. *P<0.05, **P<0.01, ***P<0.001. These *P*-values are based on a z-test statistic derived from a Maximum Likelihood Structural Equation Model using robust standard errors and a Satorra–Bentler scaled test statistic. See ref. 6767 for further details.

We first fit a mediation model where WMI in the FM and GMV in BA10 simultaneously mediate age-related differences in fluid intelligence. This model, shown in Fig. 7a, fits the data well (*χ*^2^=23.410, df=12, *P*=0.024, RMSEA=0.041 (0.015–0.065), CFI=0.993, SRMR=0.021, Satorra–Bentler scaling factor=1.055), and shows that the age-related differences in fluid intelligence (c_age>fluidint_=−0.588, *Z*=−9.14, *P*<0.00001) are multiply mediated by both BA10 GMV (a1_age>BA10_=−0.199, *Z*=−4.97, *P*<0.0001, b1_BA10>fluidint_=0.156, *Z*=4.49, *P*<0.0001, a1b1_mediation_=−0.031, *Z*=−3.53, *P*<0.001) and FM WMI (a2_age>FM_=−0.77, *Z*=−27.36, *P*<0.0001, b2_FM>fluidint_=0.131, *Z*=2.29, *P*<0.05, a2b2_mediation_=−0.10, *Z*=−2.29, *P*<0.05). Computing the effect size (see Methods) yields a mediation effect of 18.2% variance explained, corresponding to a medium to large effect size. This finding is striking, since it shows that GMV and WM connectivity of the PFC make partially independent contributions in explaining age-related differences in fluid intelligence. Notably, the b1 and b2 paths (between BA10/FM and fluid intelligence, respectively) are significant in the model even though the relationship between age and fluid intelligence is already included. This shows that the associations reported here cannot be fully explained by mere co-occurrence of age-related differences in the neural and cognitive variables.

As described above, cross-sectional mediation models should be interpreted with caution^36^. However, our findings are consistent with, and extend, prior research using longitudinal analyses^37^ and lesion studies^38^ to relate frontal regions to changes in cognition. The second mediation model, which estimates the attenuation of the age-related differences in multitasking when taking into account WM in the ATR, is shown in Fig. 7b. It too fits the data well (*χ*^2^=0.515, df=1, *P*>0.47, RMSEA=0.0 (0.0–0.0), CFI=1, SRMR=0.003, Satorra–Bentler scaling factor=0.94) demonstrating that age-related differences in multitasking ability (c_age>multitasking_=−0.189, *Z*=−3.49, *P*<0.001) are mediated by WMI of the ATR (a_age>ATR_=−0.51, *Z*=−13.00, *P*<0.0001, b_ATR>multitasking_=0.155, *Z*=2.73, *P*<0.01, ab_mediation_=−0.079, *Z*=−2.63, *P*<0.01), with an effect size of 29.4% variance explained, corresponding to a large mediation effect. Again, the significant b-path in this mediation model indicates an age-independent role for the ATR in supporting multitasking performance. For both fluid intelligence and multitasking, the brain measures-of-interest mediate the age-related differences in cognition. However, the mediation effects are partial, as is the case in almost all well-powered mediation analyses^35^. The residual age-related differences in the two factors may be explained by additional brain measures, or other types of (neural, genetic or environmental) variables.

### Multigroup neural models

Finally, we explore whether the relationship between brain and behavioural factors is identical among the youngest and oldest in our sample. To do this, we create a ‘young’ and ‘old’ subset, balancing the conflicting goals of large samples for accurate estimation with maximal age-differences across groups. We implement a common guideline of having 10 participants per free parameter estimated in the model^15^. This yields 210 young (*M*=34.76, s.d.=7.35) and 210 old (*M*=74.16, s.d.=6.48) participants with an equal age-range (Levene’s test for homogeneity of variance, *P*>0.05) for the model shown in Fig. 4. First, we fit the full model without any constraints, showing it fit this data well (*χ*^2^=65.222, df=48, *P*=0.05, RMSEA=0.041 (0.003–0.065), CFI=0.974, SRMR=0.038, Satorra–Bentler scaling factor=1.009). We then fit the model for both young and old by separately, imposing equality constraints on both (i) the behavioural factor loadings to ensure measurement invariance and (ii) the brain–behaviour relationships (but allowing the means to differ). Next, we use modification indices to ask the following question: which brain–behaviour relationships, if any, would significantly improve model fit if we were to allow them to differ in young and old? This comparison showed that two fixed parameters in the constrained model would significantly improve model fit if estimated freely. Specifically, the influence of both ATR (*χ*^2^_diff_=6.044, df=1, *P*<0.05) and FM (*χ*^2^_diff_=5.84, df=1, *P*<0.05) on multitasking is more positive in the older group when estimated freely. Notably, no such difference was observed for GMV. However, we note that although the age variance in both groups is equal, the variance in behaviour (Levene’s test for homogeneity of variance *P*<0.0001 for both fluid intelligence and multitasking) and WMI (Levene’s test for homogeneity of variance <0.0001 for both ATR and FM), but not in GMV, also increases significantly in the elderly (see Raz and Lindenberger^25^, for a discussion of the importance of increasing variance with age). This leaves open the possibility that the greater importance of ATR is due to greater variance to be explained, reorganization or a combination of both.

## Discussion

A key question in the cognitive neuroscience of aging is whether aging represents a single- or multi-factorial process^2^,^18^. Our findings show that even for two relatively similar executive functions and four exclusively frontal brain measures, unifactorial models of prefrontal aging, such as the frontal lobe hypothesis^7^ or the disconnection hypothesis^39^, are oversimplifications. A single-factor model of the four PFC variables fits poorly, and these four neural variables show distinct age-related differences and specific, differential, predictions of two cognitive factors. Second, we show that GMV in BA10 predicts individual differences in fluid intelligence beyond prefrontal WMI, showing that disconnection alone is unlikely to explain age-related differences in fluid intelligence. We further extend the role of WM connectivity in mediating age-related cognitive decline, by showing that, for multitasking, WMI becomes increasingly important for preserved cognitive function with advancing age. A related single-factor model of ageing that we do not explicitly address here is the ‘processing speed’ hypothesis^40^, which suggests that age-related slowing in mental processing underlies decline on a diversity of cognitive tasks. However, we note that, if an age-related decline in processing speed was the only underlying cause of cognitive ageing in executive functions, we would not expect to observe the complex multifactorial pattern of age-related differences described here.

Although we show considerable age-related differences in both executive functions, we also note that the specificity of the findings here offers avenues for potential interventions. Recent work has increasingly shown evidence for plasticity induced by various interventions extending into old age, which affect WM, GM and the interplay between brain structure, brain activity and complex cognitive functions^41^. The present findings raise the possibility that targeted interventions might offer more selective improvement in cognitive skills.

Fluid intelligence lies at the heart of psychometric analyses of cognitive abilities^9^, with strong statistical relationships with other higher cognitive abilities. Here we replicate the common finding of a strong age-related decline in fluid intelligence, but furthermore show that these age-related differences are multiply mediated by two PFC properties, GMV of BA10 and integrity of FM WM, that together explain 47.1% of individual differences in fluid intelligence scores. This provides evidence for models of PFC function that suggest key roles in integrating information for BA10 (ref. 42) and cross-hemispheric communication for FM^43^. Recent studies in patients with focal brain lesions have linked impaired fluid intelligence to damage in both MD regions^38^ and anterior frontal cortex^44^, suggesting an important role for both. In our data, despite strong correlation of GMVs in the two regions, it was BA10 that was the stronger predictor. Our multimodal approach thus suggests that preserved fluid intelligence in old age depends both upon the complex information processing in BA10 and on the successful interhemispheric integration of those processes by means of an intact FM. Moreover, the partially independent contributions of GMV and WMI suggest a multifactorial role in individual differences, such that people with relatively poor WMI might have comparable fluid intelligence if complemented by having greater GMV, and *vice versa*. In addition, we observe an age-related increase in inter-individual variability in the behavioural scores for both cognitive dimensions, which is compatible with prior empirical and theoretical work^25^. Importantly, our brain data suggest that one mechanism contributing to this behavioural phenomenon is likely to be the differential rate of ageing of frontal lobe systems.

In contrast to fluid intelligence, age-related differences in multitasking ability are significantly mediated by a distinct WM structure, the ATRs, a structure also known to be important for performance on complex, timed tasks^45^. Though older adults show consistent impairments in multitasking, these effects are less pronounced than differences in fluid intelligence. It is known that ageing increases the dependency on external cues (or environmental support) compared to internally generated cues. Moreover, performance of impaired adults on multitasking has been shown to improve by the addition of trivial external cues^46^, suggesting that a failure to internally cue the key cognitive steps (switching to other subtasks at the appropriate time) might be a possible mechanism of age-related differences in multitasking. However, recent work^47^ suggests that increased age-related dependence on external cues is not deterministic, but instead varies across tasks and likely across individuals, such that successfully aging individuals can, under certain circumstances, switch to increased reliance on internal cues, diminishing age-related performance differences. This suggests that our finding of a more positive influence of frontal WMI in older adults, most notably the ATR, may represent a possible structural correlate that underlies this ability to shift from external to internal cues^47^, (page 6). This could be interpreted as a form of neurocognitive scaffolding^48^, although testing this hypothesis would require further structural and functional investigation. Together, these findings suggest that ATR may be fundamental in the automated generation of time-related internal cues relevant for high multitasking performance by facilitating communication between the thalamus and PFC.

In summary, we have examined the relationship between four prefrontal structures and age-related differences in two related yet distinct executive functions in a large and unique new cohort of population-based individuals across the adult lifespan. Our results show that cognitive tasks within the domain of executive function can be differentiated, and that their decline across lifespan is multiply-determined by specific patterns of GMV and WMI, within a number of sub-regions of PFC. These results provide evidence against the dominant, unitary accounts of age-related prefrontal brain atrophy and cognitive decline, revealing instead a new perspective, where distinct prefrontal systems make independent contributions to executive functioning. Multimodal neuroimaging combined with psychometric models will allow us to investigate the most promising targets for ameliorating age-related differences in executive functions.

## Methods

### Sample

A healthy, population-based sample (*N*=610) was collected as part of the Cam-CAN. Exclusion criteria included low Mini Mental State Examination (MMSE) (≤24), poor hearing (failing to hear 35 dB at 1,000 Hz in either ear), poor vision (<20/50 on Snellen test), poor English knowledge (non-native or non-bilingual English speakers), self-reported substance abuse and current serious health conditions (for example, self-reported major psychiatric conditions, current chemo/radiotherapy or a history of stroke). We also excluded people who were not appropriate for magnetic resonance imaging (MRI) or magnetoencephalography scanning, which included those with safety- and health-related contraindications (for example, disallowed implants, pacemakers, recent surgery or any previous brain surgery, current pregnancy, facial or very recent tattoos or a history of multiple seizures or fits) as well as those with comfort-related issues (for example, claustrophobia or self-reported inability to lay supine for an hour). Only participants who underwent all structural scans and performed all behavioural tests without equipment failure were included, resulting in *N*=571 in the final sample (mean age 54.5, s.d.=18.14, range 18.46–88.9 at the time of first contact, 287 female). Outliers were defined as region-specific GM or WM values with *z*-scores exceeding 4 or −4 and were removed (*N*=4) prior to further analysis, leaving *N*=567, 286 female participants. All participants took part in a range of psychological tests (no other cognitive domains were analyzed in the context of this analysis). Ethical approval for the study was obtained from the Cambridgeshire 2 (now East of England—Cambridge Central) Research Ethics Committee. Participants gave full informed consent. *P* values for specific tests reported are corrected for multiple comparisons by means of False Discovery Rate at 0.05.

### Behavioural tasks

We assess fluid intelligence using the Cattell Culture Fair Test^49^, consisting of pencil-and-paper subtests that yield four summary scores (series completions, odd-one-out, matrices and topology) used in further modelling. We administered Scale 2, Form A, according to the standard protocol. This consisted of four subtests yielding a sum score each. In contrast to the Cattell test of fluid intelligence, the Hotel test^46^ simulates a hotel work environment and measures the ability to distribute performance across multiple tasks, which we will refer to from here on as multitasking. Figure 1 shows both tasks. For the Hotel task, participants were asked to spend a total of 10 min performing five different tasks in a simulated hotel administration environment, where each individual task would take at least 10 min to complete. Participants were instructed to spend as much time as possible on each of the tasks. This task requires goal maintenance, cognitive control and task shifting abilities in the sense that participants who became too absorbed in one task at the expense of others would score lower (2 min per task represents optimal performance). Variables-of-interest are how many of the tasks people performed in the 10-min interval (with a range between 1 and 5, with 5 being optimal) and the total time misallocated (defined as the summed deviation of the optimal performance of 120 s per task, ranging from 0 to 960). Although other tasks of multitasking are available^13^, recent work suggests that this more naturalistic setting of multitasking has greater ecological validity^50^, and provides greater sensitivity in detecting real-life changes in such abilities in clinical conditions such as dementia, bipolar disorder attention deficit hyperactivity disorder^50^,^51^ than more traditional, lab-based cognitive tests of multitasking.

### Neural measures

The first neural variable is GMV in frontopolar BA10. BA10 has been found to be active during a wide range of cognitively demanding tasks^42^, particularly those that require retaining multiple rules of a task^52^. Lesions in BA10 have been associated with declining performance both in multitasking^14^,^53^ and general intelligence^44^. The second (non-overlapping) neural variable is mean GMV within the frontal section of the MD system. This system comprises a set of distributed frontoparietal regions that include the superior and middle frontal gyri, and is known to be active across a wide range of cognitively demanding tasks^54^, and whose damage is selectively associated with reduced fluid intelligence^38^. We here focus on the frontal subset of MD regions (see Methods section for further details), comprising the presupplementary motor area, bilateral frontal operculi and bilateral dorsolateral PFC.

Recently, a growing number of papers have begun to view WM differences in the frontal cortex as the main cause of age-related differences in executive functions^17^. Two WM tracts form dense interconnections between frontal regions and to the rest of the brain, and likely play an important role in coordinating the function of these regions. The FM represents the interhemispheric pathway connecting left and right BA10 through the genu of the corpus callosum. WMI of the FM has been associated with better performance in higher cognitive faculties relevant to fluid intelligence and multitasking, such as problem solving^55^, set shifting^43^ and cognitive control^56^. Conversely, the ATR (also known as the frontostriatal tract) connects the thalamus to anterior prefrontal cortices including the anterior superior frontal gyrus, the anterior middle frontal gyri and BA10. Thus the frontal terminus of this classically defined WM tract has considerable connectivity with the prefrontal component of the MD network. Recent work^57^ in ageing populations has shown that WMI in the ATR is strongly related to scores on an executive function factor. Similarly, WMI of the ATR has been related to both processing speed and general intelligence^20^ and performance on a complex, timed task^45^. Figure 3 shows all four regions-of-interest (ROI).

### Imaging parameters and image preprocessing

The MRI data were collected from a Siemens 3 T TIM TRIO (Siemens, Erlangen, Germany). GM was estimated from a 1-mm-isotropic T1-weighted 3D Magnetization Prepared RApid Gradient Echo (MPRAGE) sequence (repetition time (TR) 2250, ms, echo time (TE) 2.98 ms, inversion time (TI) 900 ms, 190 Hz per pixel; flip angle 9°; field of view=256 × 240 × 192 mm; GRAPPA acceleration factor=2), using the SPM8 software (Wellcome Department of Imaging Neuroscience, London, UK), release 4537, implemented in the AA 4.0 pipeline ( https://github.com/rhodricusack/automaticanalysis). A unified segmentation-normalization approach (‘New segment’) employed tissue class prior probability maps for GM, WM, cerebral-spinal fluid, skull, soft tissue and remaining non-brain voxels^58^. A first-pass bias correction was implemented by an initial run of the segmentation routine. The resulting bias-corrected image was then re-segmented using a sampling distance of 1 mm and a segmented voxel size of 1.5 mm. This produced a posterior probability map for each tissue class in the native image space, as well as a posterior probability map for GM in a standard Montreal Neurological Institute (MNI) space. The voxel intensities in this MNI GM image were multiplied by the determinant of the Jacobian transformation matrix that maps from the native to MNI space, to convert from GM probability to GMV. The GMV for each ROI was then obtained simply by averaging over all voxels within that ROI. BA10 was derived from a standard MNI atlas^59^ and the temporal pole was derived from the Harvard–Oxford probabilistic atlas^60^. The MD mask was created by averaging contrasts that isolated cognitive demand across 7 diverse tasks and 40 individuals (data from ref. 54). To create a symmetrical volume, data from left and right hemispheres were averaged then projected back to both hemispheres; the volume was smoothed with a 4-mm full width at half maximum Gaussian kernel and thresholded at a value of *t*>1.5. We then defined the final MD mask for the purpose of studying PFC structure by averaging GMV across a (frontal) subset of regions comprising presupplementary motor area, bilateral middle frontal gyri, bilateral frontal operculi and bilateral dorsolateral PFC. This mask is shown in Fig. 2.

To estimate WMI, diffusion-weighted images were acquired in 64 directions with 2 averages, with TR=6.5 s, TE=93 ms, b=1,000 s mm^−3^ and GRAPPA parallel reconstruction (acceleration factor=2). Each volume consisted of 48 slices in the intercommissural plane, 2.5 mm thick with 0.5 mm gap, with an in-plane resolution of 1.8 mm and field of view=230 × 230 mm. Diffusion data was acquired on the same scanner with a twice-refocused-spin-echo sequence, with 30 diffusion gradient directions each for *b*-values 1,000 and 2,000 s mm^−2^, and three images acquired using a *b*-value of 0 (TE=104 ms, TR=9.1 s, voxel size=2 × 2 × 2 mm^3^, field of view (FOV)=192 × 192 mm^2^, 66 axial slices, GRAPPA acceleration factor=2). All preprocessing was completed using a combination of functions from FSL version 4.1.8 and custom MATLAB scripts. The diffusion data were pre-processed for eddy currents and subject motion using an affine registration model. After removal of non-brain tissue, a non-linear diffusion tensor model was fit to the DWI volumes. Non-linear fitting of the diffusion tensor provides a more accurate noise modelling than standard linear model fitting and enables various constraints on the diffusion tensor, such as positive definiteness^61^.

The tensor’s eigensystem was used to compute the fractional anisotropy (FA) at each voxel; FA maps were spatially normalized into a standard stereotactic space using tract-based spatial statistics^62^, with a standard WM template (the JHU FA atlas; http://cmrm.med.jhmi.edu/) as the target. Images were then smoothed with a 6 mm full width at half maximum Gaussian kernel to address possible residual errors and inter-individual variability and to ensure the normality requirements of parametric statistics were met, and then masked with a binarized version of each participants FA map, such that voxels below an FA threshold of 0.35 were not considered for further analysis. Next, to verify how different tracts contribute to different cognitive functioning, we extracted the mean FA values from the two ROIs, FM and ATRs, using the JHU white matter atlas ( http://cmrm.med.jhmi.edu/).

### Structural equation modelling

There are two common empirical approaches in research on the structural brain bases of individual differences in cognitive skills. The mass univariate approach examines the relationship between cognitive performance and a measure at each voxel or region in a structural MR image of the whole brain. For example, a cognitive variable representing a measure of executive function might be used to predict grey matter density^63^ or WMI^64^. This general approach has provided a wealth of findings and conjectures of brain and behaviour relationships that are then related to age-related declines in executive functions. However, the exhaustiveness of whole-brain analysis and potential for false positives may simultaneously be a weakness, leading to heterogeneous findings. Most importantly however, a mass univariate approach does not consider the interrelationships among the voxel estimates of the local brain properties. For these reasons, structural equation modelling is often a preferable alternative.

Using structural equation modelling, recent studies have found various relationships between global brain variables and individual differences in cognitive measures, including global WM and information processing speed and intelligence^16^,^17^,^21^, total brain volume and memory, executive function and verbal fluency^21^, global brain shrinkage, fluid intelligence and information processing^22^ and total cerebral blood flow and intelligence^21^. However, as powerful as these global factors have proved to be, an emerging debate has focused on the possibility that the covariance of neural properties in the (aging) brain cannot be fully explained by single global factors^18^,^34^. This suggests that modelling of more specific neural properties may be necessary to inform neurocognitive theories of ageing.

Some studies measure multiple cognitive tests per cognitive factor. Given the nature of our study this was not feasible, so we use model-specific subtests within well-validated tests instead. Thus, the latent variables here reflect what is common across the subtasks of the Cattell (series completions, odd-one-out, matrices and topology) and Hotel (total time misallocated and number of tasks attempted), an approach common in other types of latent variable models (for example, Item-Response theory, where individual items are modelled to estimate the latent trait). SEM can test whether observed patterns in the data are compatible with proposed causal hypotheses concerning the relationships between measured and latent variables. To best relate neural and behavioural measurements we fit a multifactor MIMIC model^33^. The MIMIC model is conceptually similar to partial least squares, another popular method in neurocognitive ageing research. However, the MIMIC approach has the benefits of explicit model fit, model comparisons and full information estimation methods (for example, Maximum Likelihood). For this reason, partial least squares is often considered a pragmatic alternative to the more principled, theory-driven SEM approach, which is why we here prefer the latter to test our *a priori* conceptualization^65^. In this (MIMIC) model, the cognitive domains are assumed to be causally dependent on structural brain properties (for a theoretical and empirical foundation of the MIMIC model for neuroimaging, see refs 34, 66). Moreover, all influence of the neural variables on behavioural performance goes via these latent variables (that is, direct paths between neural indicators and observed behavioural variables are not allowed). This model can be captured by two equations: The measurement model relates the *i*th behavioural measure, *y*_*i*_, to the *j*th latent variable, *η*_*j*_, with factor loadings *λ*_*ij*_ as shown in equation (1)


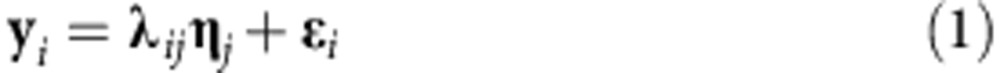


where *ε*_*i*_ refers to random measurement error in the behavioural measures.

The latent variables *η*_*j*_ in turn are assumed to be causally dependent on a weighted summation of the neural variables *x*_*k*_ weighted by *γ*_*jk*_ as shown in equation (2)


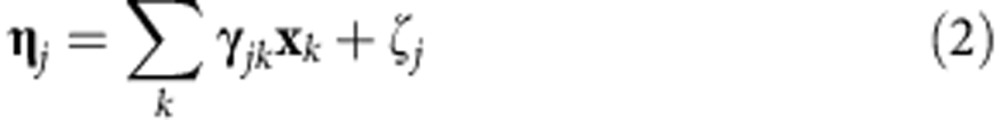


The residual term *ζ*_*j*_ captures both measurement error of the neural measurements and all residual variance in the latent variables not predicted by our neural variables. The model is fit by estimating the parameters that minimize the discrepancy between the observed covariance matrix *S* and the estimated covariance matrix Σ(*θ*), modelled as shown in equation (3)


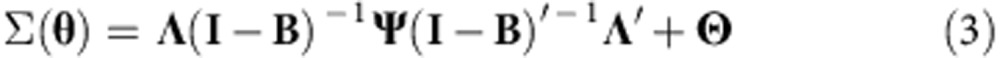


where Λ is a matrix of the factor loadings, *I* is the identity matrix of regressions, *B* is a matrix of latent regression terms, *Ψ* is the variance/covariance matrix of the latent variables and the neural predictors and Θ is a matrix of error terms. SEM were fit using the package Lavaan^67^ in R^68^, plots were generated using ggplot2 (ref. 69). We used the following guidelines for judging good fit: RMSEA<0.05 (acceptable: 0.05–0.08), CFI>0.97 (acceptable: 0.95–0.97) and SRMR<0.05 (acceptable: 0.05–0.10)^32^,^70^ and report the Satorra–Bentler scaling factor for each fitted model. All models were fit using Maximum Likelihood Estimation using robust s.e., and report overall model fit using the Satorra–Bentler scaled test statistic^67^. Mediation effect sizes were computed according to guidelines in ref. 35 as follows: multiple mediation with two mediators shown in equation (4):


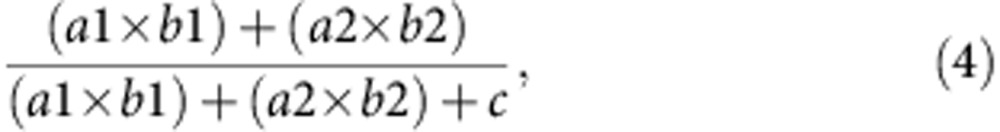


simple mediation with one predictor shown in equation (5)


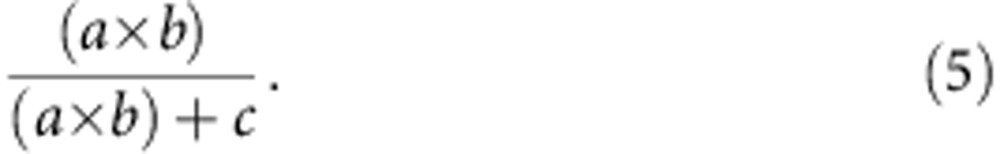


## Author contributions

R.A.K. and R.N.A.H. conceived the study. R.A.K. analyzed the data. R.A.K., S.W.D. and R.N.A.H. wrote the paper. J.R.T., S.W.D., J.D. and D.J.M. performed preprocessing analyses, conceived behavioural measurements, developed key neuroimaging pipelines and provided valuable comments on the manuscript. R.A.K. and S.W.D. created the figures.

## Additional information

**How to cite this article:** Kievit, R. A. *et al.* Distinct aspects of frontal lobe structure mediate age-related differences in fluid intelligence and multitasking. *Nat. Commun.* 5:5658 doi: 10.1038/ncomms6658 (2014).

## Supplementary Material

Supplementary Information Supplementary Figures 1-2 and Supplementary Table 1

## Figures and Tables

**Table 1:** Robust regression of age-related behavioural differences.

**Variable**	**Estimate**	**s.e.**	**Test statistic**	***P***-**value**
*Fluid intelligence and age*				
Age	−0.034	0.002	*t*-value	<2e−16
			−21.070	
Age (robust sandwich)	−0.037	0.002	*z*-value	<2e−16
			−20.831	
				
*Multitasking and age*				
Age	−0.014	0.002	*t*-value	<1.66e−11
			−6.874	
Age (robust sandwich)	−0.018	0.002	*z*-value	<4.46e−11
			−6.588	

Robust regression using heteroscedasticity consistent sandwich estimator to regress cognitive factor scores on age. Results show highly similar results for robust and regular regression, suggesting that the increase in behavioural variance with age does not affect our estimate of age-related differences in the two cognitive factors.

*P* values for the robust regression are computed using a heteroscedasticity-consistent robust sandwich estimator.
