# Supplementary material for: Distinct aspects of frontal lobe structure mediate age-related differences in fluid intelligence and multitasking
Source: Nat Commun. 2014 Dec 18;5:5658. doi: 10.1038/ncomms6658 (PMC4284640; doi:10.1038/ncomms6658)
Supplement: Supplementary Information — Supplementary Figures 1-2 and Supplementary Table 1 [file ncomms6658-s1.pdf]

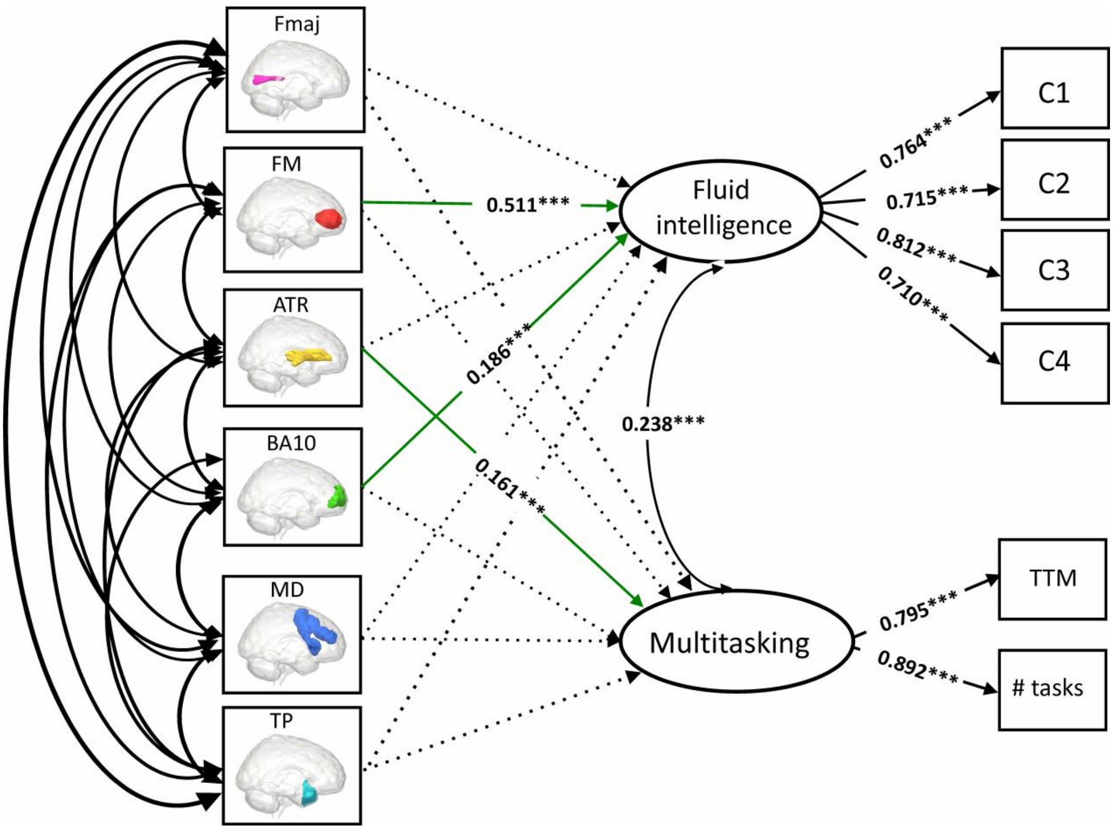

Supplementary Figure 1. Full model including two control regions

In our full model (Figure 4), we selected four regions of interest that, based on the literature, should predict our cognitive domains of interest. To further support these findings we refit an extended version of the model, adding a white matter tract and a grey matter region that we hypothesize should not predict our domains. The additional white matter tract is the Forceps Major, or the splenium of the corpus callosum, shown above. This connects early visual regions, and as such should not be expected to contribute to higher executive function. The additional grey matter region is the temporal pole, corresponding to Brodmann area 38. We then refit a model similar to that shown in Figure 4, but with these two regions added. As can be seen in Supplementary Figure 1, none of the four new brain-behavior paths for these new regions are significant. More importantly, fixing any of these four parameters to zero does not adversely affect model fit (fixing all four to 0 simultaneously: $\chi 2_{diff=}5.28$, $df_{diff}=4$, $p>.2$). In contrast, fixing any of the three key brain-behavior parameters in Figure 4 to 0 induces

significantly poorer fit. This control analysis further supports the specificity of the frontal regions we selected to figure in our model.

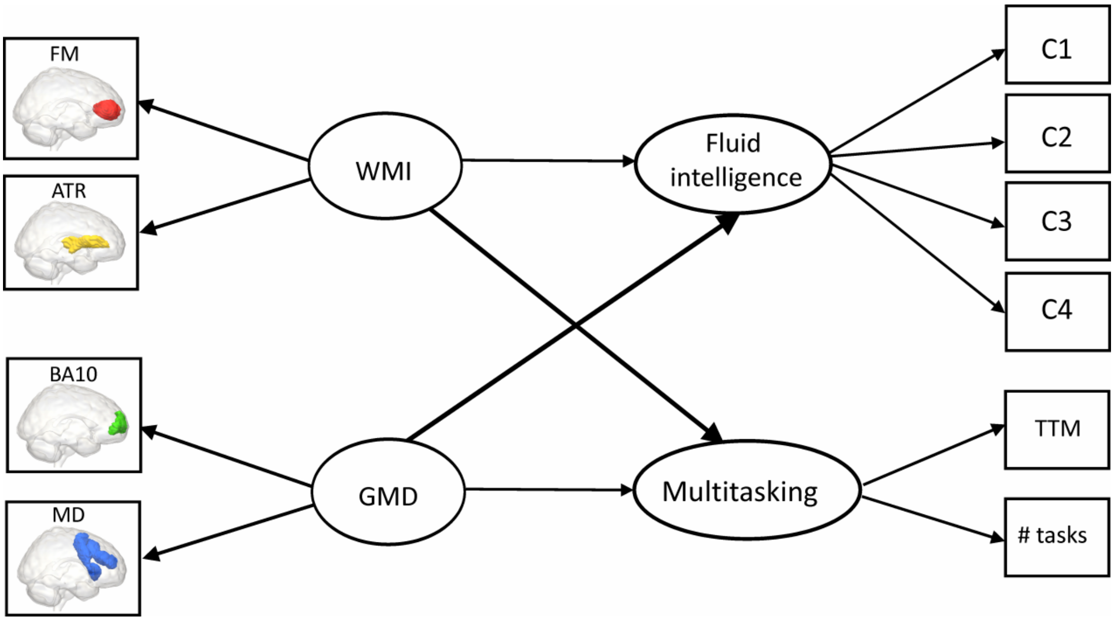

Supplementary Figure 2. Neural latent variable model

This model captures a single latent variable for grey matter density (GMD) and White matter integrity (WMI). Although it fits considerably better than the frontal model above, it fits significantly more poorly than the full model shown in Figure (AIC_{diff}=41.915; $\chi 2$= 75.699, df=29, $p<0.00001$, RMSEA= 0.053 [0.039-0.068], CFI=0.98, SRMR= 0.034, Satorra-Bentler scaling factor =1.034).

Supplementary Table 1: Full covariance matrix (Related to Main figure 3, 4 and 6).

The full variance/covariance matrix used to fit the full models in Figure 4, Supplementary Figure 1 and Supplementary Figure 2. The last two columns are only used in the analysis shown in Supplementary Figure 1.

	Cattell1	Cattell2	Cattell3	Cattell4	Hotel1	Hotel2	MD	BA10	FM	ATR	TP	FMaj
Cattell1	0.9899179	0.5355801	0.6181675	0.5574727	0.2281123	0.1985507	0.1286876	0.1646565	0.4309867	0.3258916	0.0774996	0.3350456
Cattell2	0.5355801	0.9981061	0.5779753	0.5132384	0.1776466	0.1897263	0.2017333	0.2253757	0.4102434	0.3284287	0.0768196	0.2957336
Cattell3	0.6181675	0.5779753	0.9814426	0.5420693	0.2647653	0.2610074	0.1911416	0.1945152	0.4940918	0.3718298	0.0945096	0.3500713
Cattell4	0.5574727	0.5132384	0.5420693	0.9890418	0.1240352	0.1294033	0.2812936	0.2810946	0.4141927	0.3197988	0.2024624	0.2979994
Hotel1	0.2281123	0.1776466	0.2647653	0.1240352	1.0028175	0.7109082	0.0135508	0.0418387	0.1813043	0.2056765	0.0301793	0.1911385
Hotel2	0.1985507	0.1897263	0.2610074	0.1294033	0.7109082	1.0010347	0.1002115	0.1225655	0.1858467	0.2005331	0.0790569	0.1490344
MD	0.1286876	0.2017333	0.1911416	0.2812936	0.0135508	0.1002115	0.8826314	0.7207605	0.1381533	0.1712148	0.6859622	-0.029443
BA10	0.1646565	0.2253757	0.1945152	0.2810946	0.0418387	0.1225655	0.7207605	0.9144438	0.1310856	0.1960668	0.6132722	-0.050534
FM	0.4309867	0.4102434	0.4940918	0.4141927	0.1813043	0.1858467	0.1381533	0.1310856	0.9533094	0.6981139	0.0156746	0.7150599
ATR	0.3258916	0.3284287	0.3718298	0.3197988	0.2056765	0.2005331	0.1712148	0.1960668	0.6981139	0.9120771	0.1030524	0.5595368
TP	0.0774996	0.0768196	0.0945096	0.2024624	0.0301793	0.0790569	0.6859622	0.6132722	0.0156746	0.1030524	0.8903443	-0.072686
FMaj	0.3350456	0.2957336	0.3500713	0.2979994	0.1911385	0.1490344	-0.029443	-0.050534	0.7150599	0.5595368	-0.072686	0.8443023
